# Multiscale and luminescent, hollow microspheres for gas phase thermometry

**DOI:** 10.1038/s41598-017-18942-2

**Published:** 2018-01-12

**Authors:** Lothar Bischoff, Michael Stephan, Christina S. Birkel, Christian F. Litterscheid, Andreas Dreizler, Barbara Albert

**Affiliations:** 1 Eduard-Zintl-Institute of Inorganic and Physical Chemistry, Technische Universität Darmstadt, 64287 Darmstadt, Germany; 2 Institute of Reactive Flows and Diagnostics, Technische Universität Darmstadt, 64287 Darmstadt, Germany

## Abstract

Recently developed laser-based measurement techniques are used to image the temperatures and velocities in gas flows. They require new phosphor materials with an unprecedented combination of properties. A novel synthesis procedure is described here; it results in hierarchically structured, hollow microspheres of Eu^3+^-doped Y_2_O_3_, with unusual particle sizes and very good characteristics compared to full particles. Solution-based precipitation on polymer microballoons produces very stable and luminescent, ceramic materials of extremely low density. As a result of the – compared to established template-directed syntheses – reduced mass of polymer that is lost upon calcination, micron-sized particles are obtained with mesoporous walls, low defect concentrations, and nanoscale wall thicknesses. They can be produced with larger diameters (~25 µm) compared to known hollow spheres and exhibit an optimized flow behavior. Their temperature sensing properties and excellent fluidic follow-up behavior are shown by determining emission intensity ratios in a specially designed heating chamber. Emission spectroscopy and imaging, electron microscopy and X-ray diffraction results are presented for aerosolizable Y_2_O_3_ with an optimized dopant concentration (8%). Challenges in the field of thermofluids can be addressed by combined application of thermometry and particle image velocimetry with such hollow microparticles.

## Introduction

New hollow and multiscale structures of materials for sustainable energy consumption and other applications are very much sought after^1^, but their bottom-up synthesis is challenging when self-organization^2^ or Kirkendall-diffusion fails^3^. Reports on nanosized materials are frequent^4^, but microstructures may be even more promising for certain applications like gas phase thermometry or the microencapsulation of drugs, cosmetics, inks, pigments, and chemical reagents^5^. Recent successes on closing the size-scale gap relied on photolithography or additive manufacturing as well as on the pyrolysis of polymeric microlattices, to create three-dimensional microarchitectures of TiN ceramics^6^ or Ni-P metamaterials^7^, just to name two examples. Bioinspired materials with hierarchical structures have been obtained also by freeze casting^8^. Hollow structures promise extraordinary functionalities, carbon for example is discussed for the superior lightweight mechanics of its nanolattices^9^ and the excellent catalytic activity of bimetal nanoparticles in its hollow nanospheres^10^. It is important to emphasize that while there is a significant lack in the synthesis methodology for hollow particles on the *microscale*, very interesting *nano*particles of phosphor materials have been described, e.g. SrAl_2_O_4_:Eu^2+^^,^^11^ or MOF@Fe_3_O_4_/SiO_2_^12^. Even hollow nanophosphors were synthesized, examples being Gd_2_O_2_SO_4_^13^ or SrZrO_3_:Eu^3+^ for thermometry applications^14^.

It is of high practical relevance to meet the challenge of measuring at the same time gas-phase temperature and velocity flows to investigate turbulent mixing phenomena, as discussed in a very recent and extensive review on thermographic phosphor particles^15^. Now, a new type of tracer particles of phosphor materials with an excellent thermal and fluidic follow-up behavior was developed for this purpose and is presented here, both in terms a) of synthesis and characterization and b) of applicability in thermometry (proof-of-concept). One of the major challenges in thermofluids poses the ideal heat-transfer, for example in film cooling of gas turbines^16^. Thermographic phosphors are used for contact-free temperature sensing^17^. Combustion chambers can be optimized based on surface temperature measurements^18^, and earlier we showed optimized, custom-made powders of a variety of phosphor materials to be useful for further thermometry method development^19,20^. Now, to measure flow and temperature simultaneously and thus optimize gas-processes for maximum energy efficiency, aerosolized particles were probed for combined thermometry and particle image velocimetry to measure and visualize the properties of gas streams. Single-shot laser-induced spectroscopy requires phosphor particles with sufficient sensitivity and excellent fluidic follow-up properties, meeting the challenge to yield a very good signal-to-noise ratio and sufficient temperature resolution on a microsecond timescale. For this purpose, particles were specially designed concerning their fluidic following behavior, temperature adaption, and emission intensity. We predicted low-density microscale powders to be optimal for particle image velocimetry and developed a new synthesis route to hollow microspheres of crystalline phosphor materials with good stability and efficiency at high temperatures. We were aiming at diameters of several micrometers to obtain good following behavior, a considerably smaller wall-thickness for fast temperature adaption, and high crystallinity to yield high emission intensity. Y^2^O^3^:Eu is an excellent model system since it is a well-established red emitting phosphor^21,22^ with high brightness and good thermal as well as mechanical stability. It has been prepared by many different methods (template directed synthesis^22^, Pechini sol-gel process^23,24^, precipitation methods^25^, microemulsion^26^ and spray pyrolysis^27^), but never as microscale and hollow particles in scalable amounts. Here we demonstrate an elegant route to hierarchically structured particles with high crystallinity. Furthermore, we provide a proof-of-concept of aerosolized particles enabling high-temperature thermometry.

**Figure 1:**
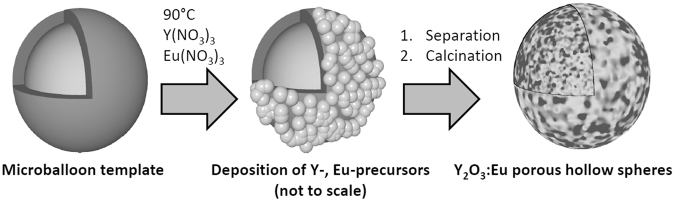
Procedure yielding hollow microspheres of Y_2_O_3_:Eu. Schematic presentation of the precipitation of Y-, Eu-precursor nanoparticles at the surface of the microballoon template, followed by calcination to form microspheres with nanoscale pores of the ceramic material.

**Figure 2:**
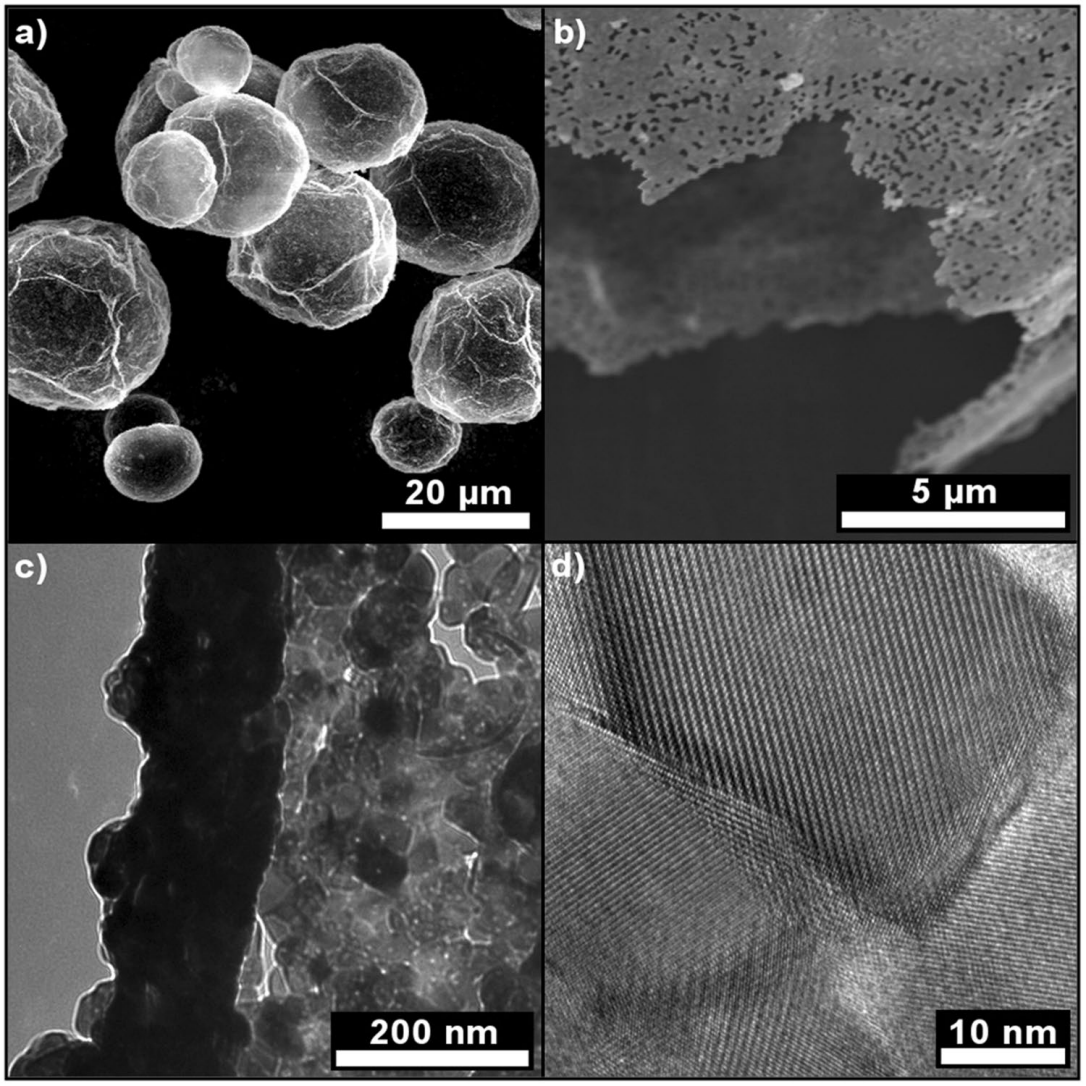
Electron microscopy study of the micro- and nanostructure of the multiscale particles. (**a**,**b**) HRSEM images of the calcined, hierarchically structured microspheres. (**c)** Top view on the cross-section of a ~150 nm thick wall shown by TEM. (**d)** HRTEM image showing the high crystallinity of the microspheres’ walls.

**Figure 3:**
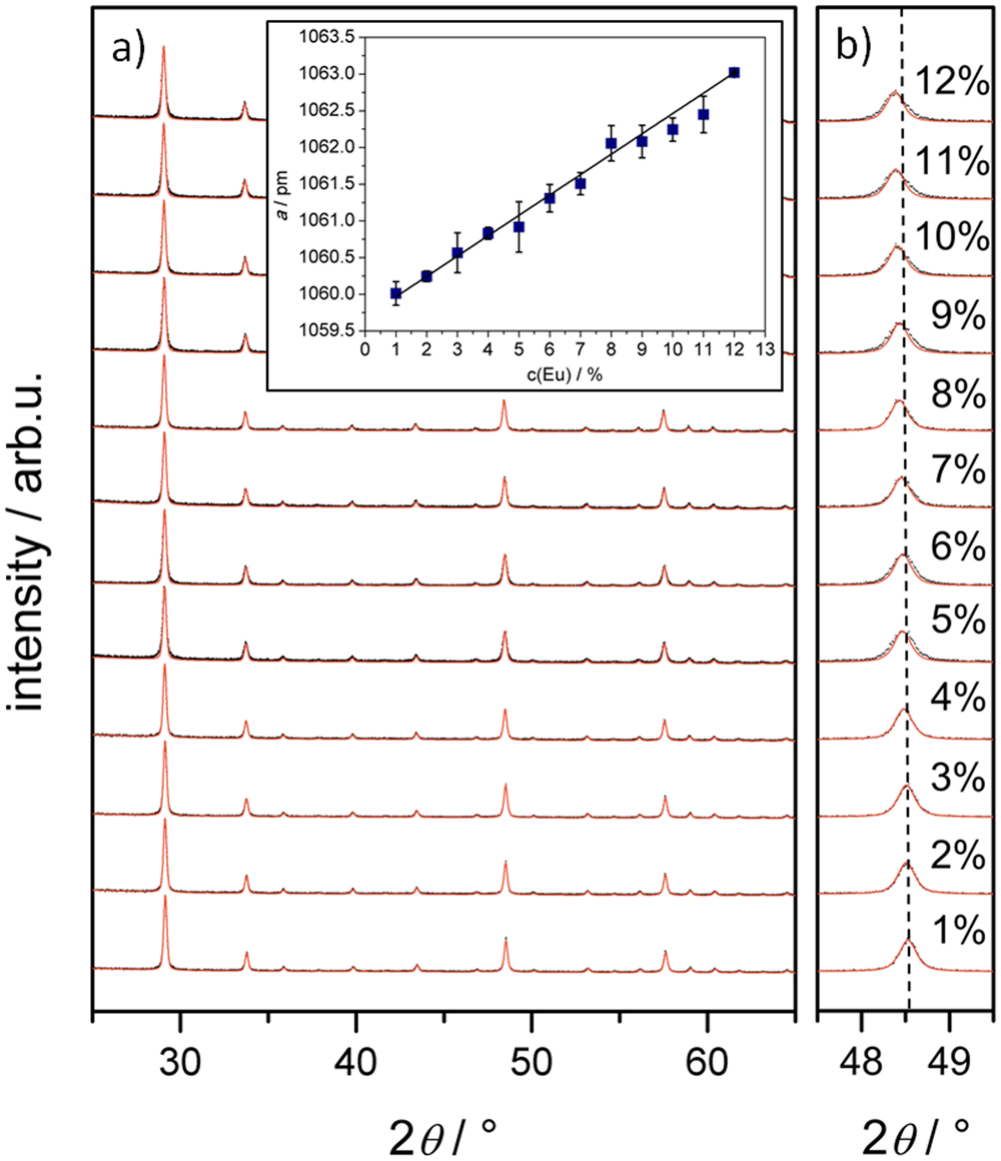
X-ray powder diffraction characterization of the Y_2_O_3_:Eu hollow microspheres. (**a)** XRD powder patterns for solid solutions with changing Eu-content. (**b)** Shifting position of the (440) reflection. Inset: lattice parameter *a* following Vegard’s law with the variation of dopant concentration *c*. Lines are meant to guide the eye.

The synthesis consists of a one-step template-directed urea-based precipitation followed by calcination as illustrated in Fig. 1. The calcination step comprises the pyrolysis of the polymer template. Polyacrylate microballoons (mean diameter is 24 µm, see Supplementary Figure S1) were treated with a urea-containing solution of Y- and Eu-nitrates for three hours at 363 K and then washed and dried. An Y- and Eu-containing precursor material was deposited on the surface of the template in form of nanospheres. Un-reacted templates were easily separated from the reaction mixture. Then, precursor-coated polymer particles were filtered from the solvent and heat-treated. After calcination at 723 K (12 h) and 1273 K (5 h) in air, hollow microspheres of a ceramic material following the size distribution of the template (see Supplementary Figure S1) were obtained. Thermolysis of the polymer and calcination of the phosphor occur simultaneously, and a powder consisting of non-agglomerated, crystalline hollow spheres of Y_2_O_3_:Eu is formed. The unconventional template plays a crucial role in the process. Compared to full templates, the hollow, micrometer-sized polymer balloons with a very low-density (24 kg.m^−3^) cause low carbon contents of the precursor materials and low CO_2_ losses during thermolysis. This results in very small, nanoscale pores (mesopores according to IUPAC^28^) in the ceramic walls that enhance the stability and crystallinity of the thermographic microspheres, which is both very important for the envisioned use in particle image velocimetry. High-resolution transmission (HRTEM) and high-resolution scanning electron microscopy (HRSEM) images exhibit hollow microspheres (Fig. 2) with a mean diameter of 25(±14) µm and a wall-thickness of less than 200 nm. The synthesis was easily up-scaled from five milligrams of product to 400 mg. Powder X-ray diffraction data (Fig. 3) show the high phase-purity and crystallinity of the powder. Multiple measurements on differently doped samples were used to monitor the successful inclusion of variable contents of Eu^3+^, which is illustrated in Fig. 3 by the changes of the reflection positions resulting in increased lattice parameters.

**Figure 4:**
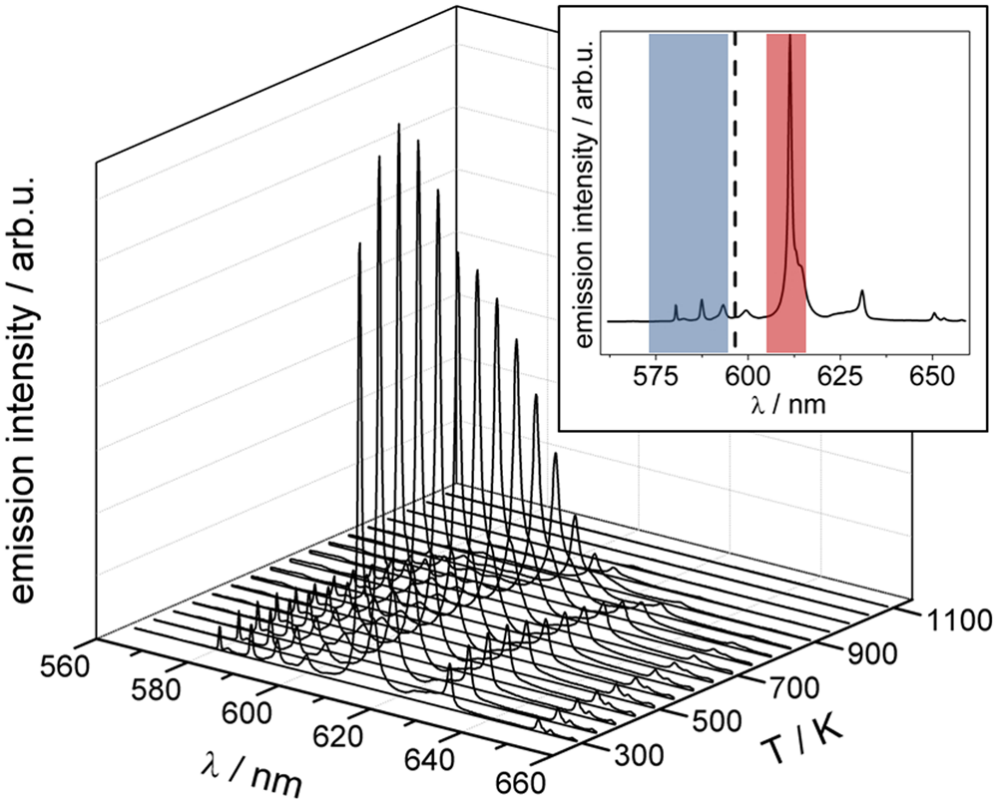
Furnace luminescence measurements on Y_2_O_3_: 8% Eu hollow microspheres. Spectra recorded at different temperatures show increasing emission intensity followed by thermal quenching. The inset provides a room temperature spectrum with the filter sets used.

By differential scanning calorimetry it was shown (see Supplementary Fig. S2), that samples of the microspheres, both as-synthesized (*c*_*p*_ = 0.43(3) J/(g·K) at 423 K) and compacted (*c*_*p*_ = 0.44(0) J/(g·K) at 423 K), had specific heat capacities comparable to those of bulk material as reported in literature (*c*_*p*_ = 0.45 J/(g·K) for undoped Y_2_O_3_^29^, see Supplementary information for further discussion). Surface determination resulted in a value of ~11 m²·g^-1^. Furthermore, all samples were subjected to energy-dispersive spectroscopy to verify the composition. A miscibility up to 12% Eu was observed, and the doping concentration of Eu optimal for thermometry was determined by luminescence measurements at room temperature under static conditions. A doping concentration of about 8% (*a* = 1062.0(2) pm, compared to *a* = 1062.2(1) pm for bulk Y_2_O_3_:8%Eu in literature^30^ exhibited the highest emission intensity. Thus, samples of hollow microspheres of Y_2_O_3_ with 8% Eu were then used to demonstrate one of the diverse possible applications of hollow microspheres: gas phase thermometry. This presumes that the microspheres rapidly adapt to the surrounding gas phase temperature. We predicted microspheres to be advantageous compared to solid particles due to their much lower thermal inertia. Because of the lower slip between velocities of the gas phase and the microspheres during rapid acceleration typical for turbulent motions, their minute mass inertia should be additionally favorable for particle image velocimetry^31^. For thermometry, the temperature-dependent spectral properties of Y_2_O_3_:Eu were exploited. Two transitions in Eu^3+^, ^5^D_0_ → ^7^F_0,1_ and ^5^D_0_ → ^7^F_2_, were used for temperature reading. The relative intensities of the spectral bands in the range of 580 to 650 nm change upon UV excitation (266 nm), as indicated in Fig. 4 (see also Supplementary Fig. S3 for a 2D presentation). Luminescence intensities in two spectral ranges (inset in Fig. 4 highlights the two spectral bands, see also Supplementary Fig. S4) were integrated prior to calculating their ratio. This ratio is temperature-dependent due to site symmetry of Eu^3+^ in Y_2_O_3_^32,33^ and can be calibrated against thermocouples.

**Figure 5:**
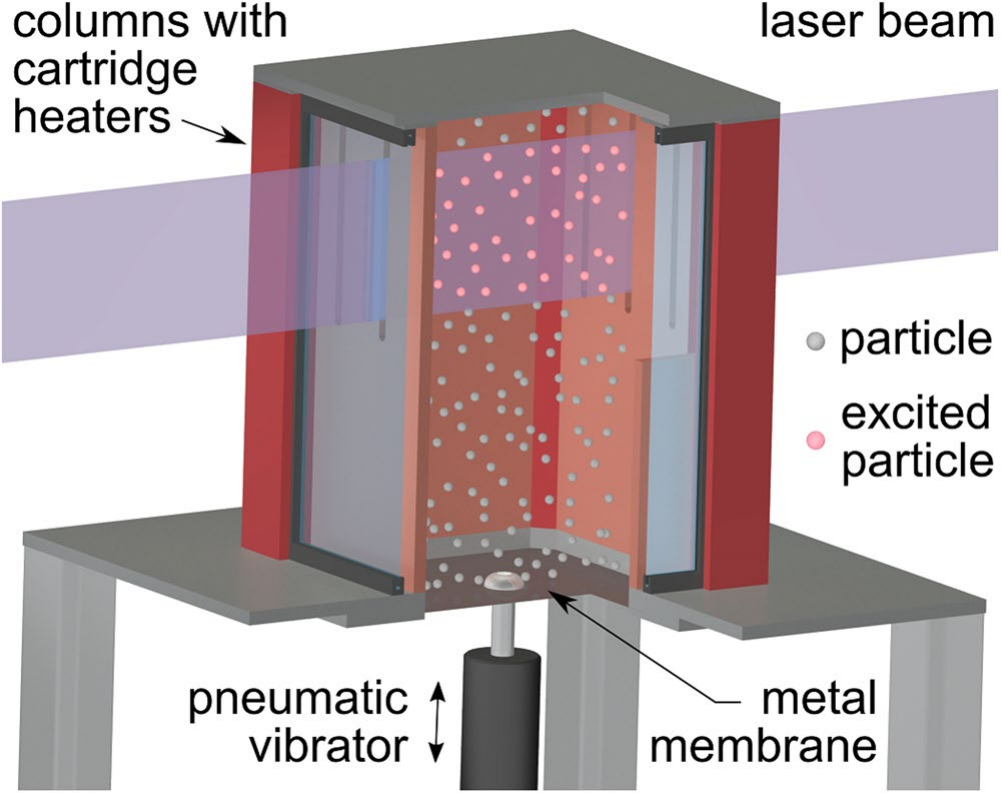
Schematic of the floating particle test rig. Heating chamber with pneumatic vibrator to aerosolize the particles. Phosphor particles are excited by a Nd:YAG laser and the emission intensity is recorded by CCD cameras.

Standard methods with aerosolized (full) particles require several hundred grams of phosphor material. Due to the restricted amounts of product available from the novel synthesis procedure at this early stage of investigations, a new and sophisticated measurement concept was developed to allow for a proof-of-concept for hollow microspheres thermometry. To demonstrate that the newly designed particles are especially suitable for this method of temperature measurement, a test rig was designed based on momentum transfer to aerosolize the microspheres into the gas phase. The working principle is outlined in Fig. 5 and more details are provided in the experimental subsection.

**Figure 6:**
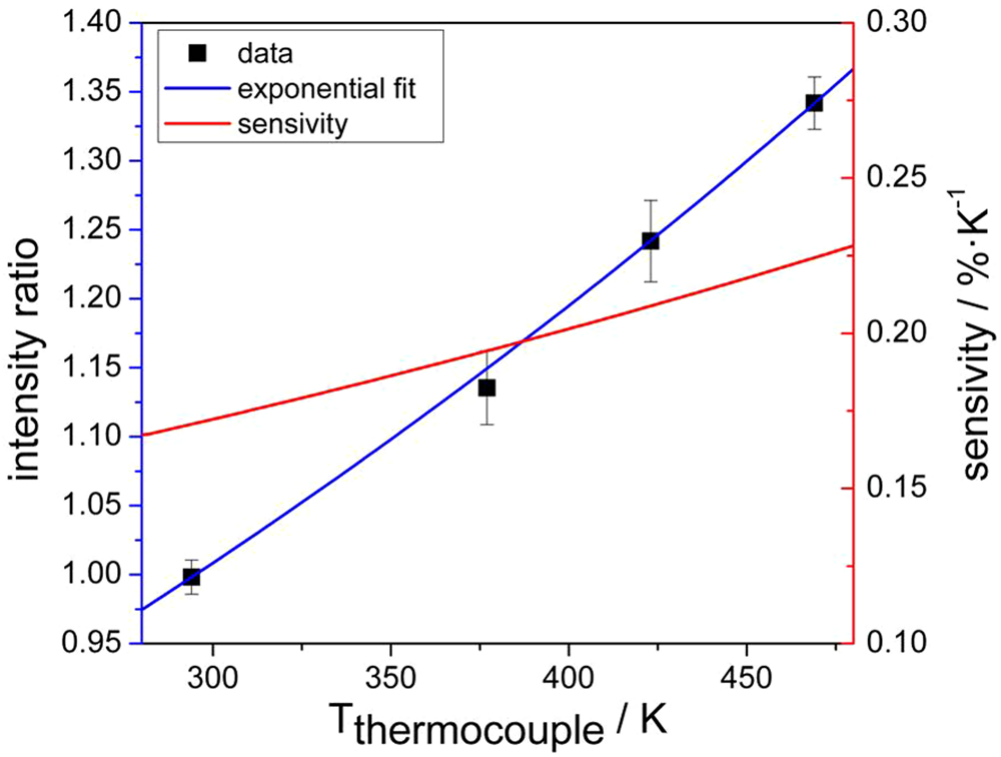
Temperatures derived by thermometry compared to thermocouple measurement. Emission intensity ratios measured with the filter set shown in the inset of Fig. 4. (blue) and sensitivities derived as described in the text (red).

Within an air-filled, heatable and optically accessible box-shaped chamber the Y_2_O_3_:Eu hollow microspheres were placed onto a metal membrane. This membrane was set into oscillation transferring momentum to the microspheres such that they float the entire volume of the chamber. Due to the low mass inertia and the low flow velocity within the chamber, the microspheres remained unsupported in air for the full period of measurement. Luminescent centers of the microspheres were excited electronically using a vertically oriented two-dimensional UV laser light sheet that intersects the chamber centrally. The luminescence emission was recorded by two CCD cameras. Each of the cameras was equipped with interference filters covering a specific spectral range (inset of Fig. 4). The filters were optimized for a best compromise between signal-to-noise ratio and high temperature sensitivity. Figure 6 shows the intensity ratio of the two spectral bands in a temperature range between 294 and 469 K as measured by thermocouples. The error bars correspond to two standard deviations obtained from 700 consecutive single laser shot measurements performed under stationary conditions of the chamber. The mean values of the ratio are well presented by an exponential fit which traces spectral properties of Y_2_O_3_:Eu luminescence back to the broadly established practical temperature standard in engineering applications. The absolute temperature sensitivity is 34% and therefore similar to other engineering applications^34,35^. The relative sensitivities in Fig. 6 are based on a calculation following equation (1) from ref.^36^, with Δ being the temperature-dependent ratio and *T* the temperature.

1 $${S}_{r}=\frac{1}{{\rm{\Delta }}}|\frac{\delta {\rm{\Delta }}}{\delta T}|$$

A theoretical calculation of the intensity ratio based on the spectra^36^ is not possible as the ratio varies significantly with changes in the optical arrangement, as shown earlier^37^.

The average temperature resolution is around 5% and is comparable with other studies^16^. Notice that the spectral properties of Y_2_O_3_:Eu microspheres hardly differ from solid particles. Furthermore, the hollow particles remained essentially un-destructed after the thermometry experiment, which was shown by additional HRSEM measurements.

With this work, we provide hollow and microscale particles of a phosphor by using an unprecedented, easy and scalable synthesis route based on hollow templates. It has to be emphasized that the range of particle diameters of approximately 25 µm is very difficult to obtain otherwise. Typical syntheses of hollow spheres are limited to products smaller 2 µm. We envision this synthesis route to be applicable to all kinds of functional ceramics with possible applications not only in thermometry but also in catalysis, controlled drug-release, or food technology.

To test our hypothesis that hollow spheres should be especially suitable for thermometry and particle image velocimetry we designed a new type of measurement set-up, enabling the microspheres to float the gas volume activated by an oscillating membrane at the bottom of a heated chamber. Due to the extremely low density of the particles they hover in air for tens of seconds and can be excited by laser light, and then analyzed. Their emissions were used for the temperature measurements. With this new design of a measurement set-up we are now able to present a very convincing proof-of-concept of our initial idea: the hollow particles can be used for an improved gas thermometry method.

The synthesis method, however, is not restricted to Y_2_O_3_:Eu, and we have already shown it to be successful for other phosphors and catalysts like those described in ref.^38^. Furthermore, it is possible to control the wall thickness and pore sizes by varying the synthesis conditions. This will be reported in a separate study.

## Methods

### Synthesis of Y_2_O_3_:Eu as hollow microspheres

Hollow particles of Y_2_O_3_:Eu were synthesized via a wet chemical urea-based deposition reaction. Hollow microballoons (Expancel 921 WE 40 d24, AkzoNobel) were chosen as templates. All other chemicals were purchased from Acros Organics and used without further purification.

In a typical synthesis, microballoons (2.5 g) were transferred into a 250 ml three-neck round bottom flask with addition of 125 ml of demineralised water. The suspension obtained was stirred and heated to 342 K. Meanwhile Y(NO_3_)_3_·6H_2_O (99.9%, 352.4 mg), Eu(NO_3_)_3_·6H_2_O (99.9%, 35.7 mg) and urea (99.5%) were dissolved in 25 ml demineralised water and were added slowly to the suspension via a dropping funnel, while the reaction temperature was increased up to 363 K. The ratio of Y and Eu was chosen according to the targeted doping concentration; the total amount added up to 1 mmol. The amount of urea was 3 g (50 mmol). The reaction mixture was stirred for 3 h and then cooled to room temperature. The suspension was transferred into a separating funnel. The precursor-coated templates were separated from small amounts of undesired bulk precipitate, then filtered in a suction filter and washed with demineralised water, ethanol, and diethyl ether, and dried. Slightly yellow precursor particles were transferred into an alumina crucible and heat-treated in air (12 h at 522 K, continued by 5 h at 1273 K, heating rate = 4 K/min). Afterwards the samples were characterized through X-ray diffraction, scanning electron microscopy and energy-dispersive X-ray spectroscopy.

### Characterization

X-ray powder diffraction data were collected at room temperature by a powder diffractometer (STOE Stadi P, linear PSD) with Cu radiation (Ge monochromator, *λ* = 1.540598 Å, flat plate sample holder, transmission geometry). For Rietveld refinement, the published structure of Y_2_O_3_^39^ was chosen as a starting model and the TOPAS suite of programmes^40^ was used.

Particle size and morphology were characterized using a high-resolution scanning electron microscope (Philips XL30 FEG, 15–30 kV acceleration voltage) equipped with an energy-dispersive X-ray spectrometer (EDS, EDAX Genesis) and a transmission electron microscope (Jeol Jem-2100F, 200 kV acceleration voltage).

Differential scanning calorimetry measurements were executed using a Netzsch instrument (DSC 404 F1 Pegasus) and platinum crucibles in an atmosphere of dried Ar.

Surface determination was performed by nitrogen adsorption-desorption at 77 K (Quadrasorb SI, Quantachrome Instruments). Data evaluation was carried out with the software QuadraWin (version 6.0).

### Optically accessible heated chamber

The optically accessible chamber was designed to aerosolize a small amount of Y_2_O_3_:Eu hollow microspheres. The box-shaped chamber has dimensions of 200 × 165 × 165 mm. The metallic frame is equipped with eight cartridge heaters (6.5 × 60 mm, 250 W, Türk und Hillinger) to vary the temperature of the chamber between 294 and 469 K. Side and top walls are either made of copper (6 mm) or quartz plates (3 mm). The high thermal conductivity of the copper plates ensures a rapid homogenization of the chamber temperature. Two opposite copper side walls are equipped with narrow quartz windows providing access for the laser radiation. Perpendicular to the axis of laser propagation a larger quartz window enables imaging of luminescence emitted from single microspheres (Fig. 5). The bottom of the chamber consists of a 0.3 mm thick metal membrane (Martin Membrane Systems). Using a pneumatic vibrator (NTS180, Netter) the membrane was set into oscillations. The pneumatic vibrator was controlled by a valve and triggered externally for synchronization with the laser pulses. The chamber is equipped with seven thermocouples distributed across the volume to monitor the apparent gas temperature and temperature homogeneity. Three thermocouples are placed in the centerline perpendicular to the field-of-view with a distance of 35 mm to each other (max. deviation 1.2 K) and four thermocouples in each corner with a distance of 10 mm to the walls (max deviation 7.2 K). Therefore, the standard deviations shown in Fig. 6 are hardly influenced by the spatial temperature distribution within the box-shaped chamber.

A sample (400 mg) of Y_2_O_3_:Eu hollow microspheres was placed on the membrane at the bottom of the chamber. The pneumatic vibrator was operated twice for one second at a frequency of 3500 Hz prior to the actual measurements. The hollow spheres remained aerosolized for several tens of seconds.

### Phosphor thermometry

Phosphor thermometry is used for measuring gas phase temperatures. This presumes a low thermal inertia of the phosphor particles. For an adequate signal-to-noise ratio, the dimensions of individual particles have to be at the micrometer scale. These two requirements motivated the use of hollow microspheres.

Following a pulsed UV laser excitation (266 nm), the emission spectrum of the phosphor Y_2_O_3_:Eu changes with temperature. For planar temperature measurements two spectral bands were monitored separately using two CCD cameras. These spectral bands were selected by appropriate bandpass filters and are denoted as “blue” (S_ph_^blue^) and “red” (S_ph_^red^) channels. For a thermographic phosphor such as Y_2_O_3_:Eu, the intensity ratio (*r*) of the “blue” and “red” filter band is a function (equation (2)) of the phosphorescence quantum yield ${\varphi }_{{ph}}$ in each channel^41^:

2 $$r=\frac{{S}_{ph}^{blue}}{{S}_{ph}^{red}} \sim \frac{{\varphi }_{ph}^{blue}}{{\varphi }_{ph}^{red}}$$

The temperature-dependent term is usually calibrated against thermocouple measurements. Spectral bands must be properly selected to combine as best as possible a high temperature sensitivity with high luminescence intensities^41^. For this purpose, the emission spectra of Y_2_O_3_:Eu hollow microspheres were recorded upon UV excitation in an electrical tube furnace using a Czerny-Turner type spectrometer in combination with a CMOS camera. As a result of these preparatory measurements, Fig. 4 shows normalized emission spectra for temperatures between 300 K and 1100 K. Using this data, two bandpass filters were selected that were optimized for temperatures ranging from 290 to 500 K (inset of Fig. 4); this interval corresponds to the conditions investigated for aerosolized hollow microspheres.

Hollow microspheres were excited by the fourth harmonic (266 nm) of a pulsed Nd:YAG laser (Q-switch operation, Quanta Ray, INDI Series) operated at 5 Hz. A combination of half-wave plate and polarizer was used to adjust the laser pulse energy to 35 mJ which was constantly monitored. Using a set of spherical and cylindrical lenses the laser beam was formed into a 40 mm high and 200 µm thick light sheet intersecting the optically accessible chamber centrally. Upon single-pulse excitation, the luminescence signal was collected perpendicularly to the laser light sheet and parallelized by an achromatic lens (500 mm focal length). Using a dichroic beam splitter (Chroma, center wavelength: 594 nm) the luminescence signal was split into its “blue” and “red” channels. Additional interference filters in front of each CCD camera monitoring both channels were centered at 580 nm and 610 nm, respectively (Chroma Technology, ET 580/25x, FWHM: 25 nm and ET 610/10 m, FWHM: 10 nm). The CCD cameras (PCO, Sensicam qe, 1376 by 1040) were equipped with a Nikkor and a Carl Zeiss Objective (both f# of 1.4, 85 mm), respectively. The field-of-view was 52 × 40 mm and a 2 × 2 pixel hardware binning was applied to reduce read-out noise. Finally, a spatial resolution of 230 µm was achieved. The camera’s exposure times were set to 2 ms, starting 10.5 µs prior to the laser excitation.

### Post-processing

Converting digital images of the “blue” and “red” channel into temperatures requires a sequence of post-processing steps.

1. Very large hollow spheres or agglomerates cause high luminescence intensities exceeding the dynamic range of the CCD cameras. Accordingly, saturated pixels were identified by their count-level and were excluded from any further analysis.
2. The background is specific to the “blue” and “red” channel and was subtracted from each image. Mean background images were recorded separately with the UV-laser in operation but without any aerosolized seeding particles.
3. The densities of aerosolized hollow microspheres varied over time. Images with too low/too high densities were excluded from further processing. Too high densities of aerosolized microspheres are prohibitive due to multiple scattering that causes systematic errors^41^. For identifying individual microspheres in a first step, an intensity threshold of 50 counts was applied to the images of the “red” and 20 counts to the “blue” channel to remove remaining background noise. Subsequently microspheres were identified as local maxima within sub-regions extending 3 by 3 pixels. If for an ensemble average of 100 individual images the number of microspheres identified in the field of view ranged between 4 × 10^10^ and 7 × 10^10^ particles/m^3^, the image sequence was accepted for further processing.
4. The images of the “blue” and “red” channels were mapped onto each other in physical space using a calibration target (La Vision, type 7) and registered by a custom-made Matlab algorithm.
5. A 7 × 7 pixel moving average filter was applied to reduce noise and to prepare for the ratio operation.
6. According to eqn. (1) the intensity ratio of the “blue” and “red” channels were calculated from the ensemble average of pairs of 700 single-shot images.
7. A 4 × 4 binning was applied to increase the signal to noise ratio.
8. Using spatially homogeneous conditions at room temperature a flat field correction was conducted to correct for vignetting and inhomogeneous CCD sensitivities.
9. A 7 × 7 pixel moving average filter was applied to smooth the ratio.
10. The resulting intensity ratio of a 1.5 mm × 1.5 mm sub-region at the center of the chamber was spatially averaged and correlated to the reading of a thermocouple (type K, 1.5 mm diameter). The thermocouple was placed prior to each measurement of the mean intensity ratio at the central position of this sub-region. A calibration curve resulted that allows to trace back the optical measurement to a well-accepted temperature standard used in engineering. Figure 6 shows the calibration curve for a temperature range spanning from 294 K to 469 K. This is a proof-of-concept showing that Y_2_O_3_:Eu hollow microspheres can replace solid phosphor particles but with much reduced thermal inertia and mass inertia.

## Electronic supplementary material

Supplementary Information
