# Supplementary material for: Multiscale and luminescent, hollow microspheres for gas phase thermometry
Source: Sci Rep. 2018 Jan 12;8:602. doi: 10.1038/s41598-017-18942-2 (PMC5766626; doi:10.1038/s41598-017-18942-2)
Supplement: Supplementary file 1 — Supplementary Information [file 41598_2017_18942_MOESM1_ESM.pdf]

Multiscale and luminescent, hollow microspheres for gas phase thermometry

Lothar Bischoff[1], Michael Stephan[2], Christina S. Birkel[1], Christian F. Litterscheid[1], Andreas Dreizler[2*], Barbara Albert[1*]

[1]Eduard-Zintl-Institute of Inorganic and Physical Chemistry, Technische Universität Darmstadt, 64287 Darmstadt, Germany, [2]Institute of Reactive Flows and Diagnostics, Technische Universität Darmstadt, 64287 Darmstadt, Germany

*albert@ac.chemie.tu-darmstadt.de, dreizler@csi.tu-darmstadt.de

Supplementary information

Size distribution

In order to determine the particle size distributions of the commercially available microballoon templates as well as the here synthesized hollow microspheres, SEM micrographs exhibiting 209 and 110 particles, respectively, were used. The resulting diameters of the single particles were obtained with the help of the program ImageJ and are presented in Supplementary Figure 1. Please note that for the unreacted template the range of diameters determined by us differs from that provided by AkzoNobel (35 – 50 μm). The size distributions of the template and the product however coincide very well.

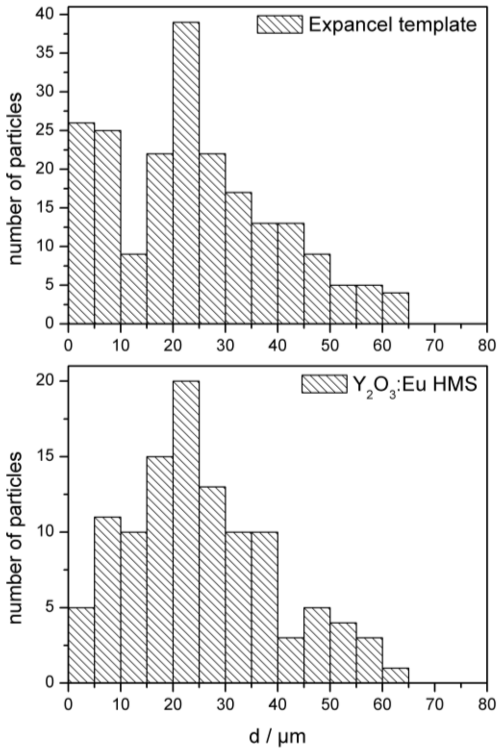

Supplementary Figure S1. Particle size distribution of the microballoon template (Expancel) and the hollow microspheres of Y_2O_3:Eu. The diameter *d* of the synthesized phosphor particles (25±14 μm) follows the measured particle size distribution of the template (24±15 μm).

Differential Scanning Calorimetry (DSC)

DSC measurements were performed in Pt-crucibles from room temperature to 1100 K under argon atmosphere. The data show similar heat capacities for both samples measured, the product as-synthesized and compacted. C_P values from literature were used to calculate the trace for bulk material (upper line in Suppl. Figure 2). The calculation was performed using the software Thermo-Calc with the SGTE Substance Database SSUB Vers. 5.2, January 2017. Samples of the microspheres, both as-synthesized (c_p = 0.43(3) J/(g·K) at 423 K) and compacted (c_p = 0.44(0) J/(g·K) at 423 K), had specific heat capacities comparable to those of bulk material as reported in literature (c_p = 0.45 J/(g·K) for undoped Y_2O_3 (V. Swamy et al., *Journal of Alloys and Compounds* 269, **1998,** 201 –207).

The calculated values of c_p of a Eu-doped bulk material are based on the Kopp-Neumann law ($c_{p,alloy}$ = sum of c_p of alloy constituents), which resulted in 0.51 J/(g·K) at 423 K. The measured and calculated values of c_p are convincingly similar, taking into account the difficulties of measuring samples of the very light hollow spheres which leads to higher inaccuracies than typical at DSC.

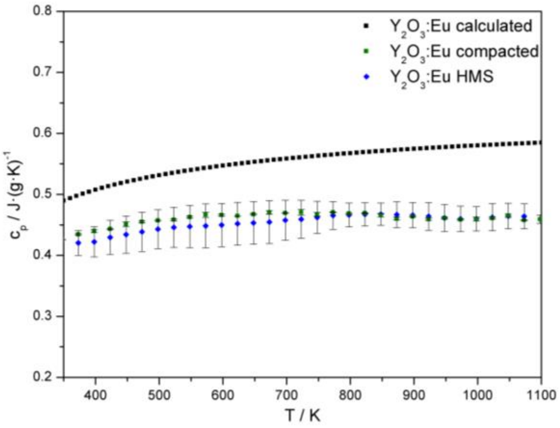

Supplementary Figure S2. DSC measurements of Y_2O_3 doped with 8% Eu. The calculated heat capacity of compacted particles and hollow microspheres are very similiar and reflect the calculated data for bulk material.

Spectroscopy

To complement the waterfall presentation in Figure 4 of the manuscript, a 2D picture is shown here (Fig. S3) and the emission intensity is compared for several bands (Fig. S4).

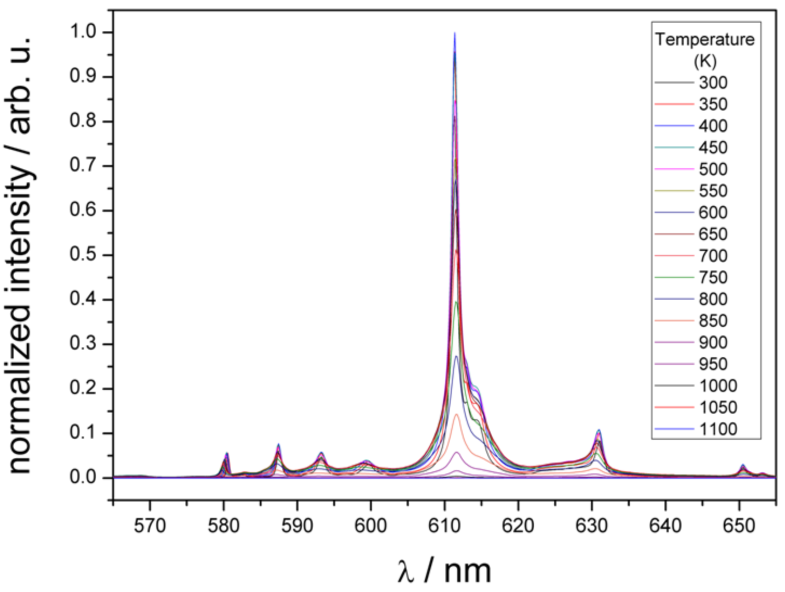

Supplementary Figure S3. Temperature-dependent emission spectra of Y_2O_3:Eu between 300 and 1100 K.

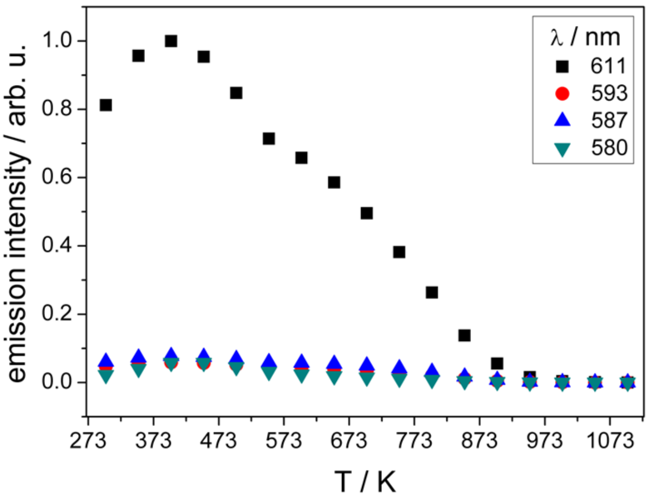

Supplementary Figure S4. Temperature dependence of the peak maxima of Y_2O_3:Eu used for phosphor thermometry.
